# Supplementary material for: Association between statin use, atherosclerosis, and mortality in HIV-infected adults
Source: PLoS One. 2020 Apr 30;15(4):e0232636. doi: 10.1371/journal.pone.0232636 (PMC7192415; doi:10.1371/journal.pone.0232636)
Supplement: S1 Table — (DOCX) [file pone.0232636.s001.docx]

**S1 Table. Weighted baseline characteristics (median, interquartile range) of HIV-infected adults recommended for statins stratified by statin use.**

|  | **Statin (-)**  **(n=93)** | **Statin(+)**  **(n=24)** | **All**  **(n=117)** | **P-value** |
| --- | --- | --- | --- | --- |
| Demographic |  |  |  |  |
| Age, years | 54.1 (49.4, 58.4) | 57.7 (50.4, 62.1) | 54.8 (49.8, 60.1) | 0.058 |
| Race |  |  |  | 0.205 |
| Caucasian | 54% | 78% | 59% |  |
| African American | 35% | 20% | 32% |  |
| Latino | 5% | 0% | 4% |  |
| Other | 6% | 2% | 5% |  |
| Gender |  |  |  | 0.482 |
| Male | 94% | 100% | 95% |  |
| Female | 5% | 0% | 4% |  |
| Transgender (M>F) | 2% | 0% | 1% |  |
| BMI, kg/m^2^ | 25 (22, 28) | 25 (23, 31) | 25 (22, 28) | 0.484 |
| SBP, mmHg | 126 (120, 136) | 124 (116, 141) | 125 ( 118, 136) | 0.821 |
| DBP, mmHg | 78 (72, 84) | 76 (72, 82) | 78 (72, 82) | 0.839 |
| HIV factors |  |  |  |  |
| HIV duration, yr | 15 (10, 20) | 16 (14, 19) | 16 (11, 19) | 0.162 |
| Nadir CD4, cells/uL | 1.8 (0.7, 3.0) | 2.8 (2.0, 3.8) | 2.0 (0.8, 3.0) | 0.121 |
| Cur CD4, cells/uL | 4.3 (2.5, 6.7) | 5.9 (4.8, 8.8) | 4.8 (3.0, 6.9) | 0.075 |
| Treated, suppressed | 47% | 71% | 52% | 0.178 |
| PI, yr | 3.1 (0, 5.7) | 2.6 (1.3, 6.0) | 2.8 (0, 5.8) | 0.414 |
| HAART, yr | 4.2 (0, 6.3) | 2.6 (2.6, 7.9) | 4.0 (0, 6.3) | 0.822 |
| Hepatitis C | 24% | 2% | 19% | 0.020 |
| Lipodystrophy | 63% | 76% | 66% | 0.232 |
| Comorbidities |  |  |  |  |
| Family history | 18% | 12% | 17% | 0.510 |
| Hypertension | 46% | 43% | 45% | 0.836 |
| DM | 13% | 10% | 12% | 0.722 |
| Any smoking | 74% | 68% | 73% | 0.595 |
| Aspirin use | 27% | 67% | 35% | <0.001 |
| Baseline CVD | 7% | 10% | 7% | 0.657 |
| Labs |  |  |  |  |
| Cholesterol, mg/dL | 192 ( 166, 217) | 203 ( 170, 253) | 192 ( 170, 222) | 0.106 |
| LDL-C, mg/dL | 111 ( 94, 139) | 91 ( 78, 174) | 111 ( 83, 140) | 0.376 |
| HDL-C, mg/dL | 40 ( 32, 49) | 42 ( 27, 54) | 41 ( 30, 49) | 0.525 |
| Triglyceride, mg/dL | 148 ( 99, 235) | 152 ( 119, 323) | 150 ( 106, 283) | 0.096 |
| hs-CRP, mg/L | 1.8 ( 0.6, 4.6) | 2.6 ( 1.1, 5.7) | 2.0 ( 0.9, 4.7) | 0.950 |
| Glucose, mg/dL | 89 ( 78, 101) | 94 ( 89, 106) | 91 ( 82, 105) | 0.428 |
| Risk |  |  |  |  |
| Fram risk factors | 3 ( 2, 3) | 2 ( 2, 3) | 3 ( 2, 3) | 0.794 |
| 10-yr Fram risk score | 10 ( 8, 14) | 12 ( 6, 12) | 11 ( 8, 14) | 0.959 |
| 10-yr ASCVD risk score | 11 ( 9, 13) | 12 ( 9, 15) | 11 ( 9, 13) | 0.291 |

­­­­­BMI = body mass index, SBP = systolic blood pressure, DBP = diastolic blood pressure, PI = protease inhibitor, HAART = highly active anti-retroviral therapy, DM = diabetes mellitus, LDL-C = low density lipoprotein cholesterol, HDL-C = high density lipoprotein cholesterol, hs-CRP = high sensitivity C-reactive protein, Fram = Framingham, ASCVD = atherosclerotic cardiovascular disease.
