# Supplementary material for: Association between statin use, atherosclerosis, and mortality in HIV-infected adults
Source: PLoS One. 2020 Apr 30;15(4):e0232636. doi: 10.1371/journal.pone.0232636 (PMC7192415; doi:10.1371/journal.pone.0232636)
Supplement: Attachment — Submitted filename: Response to Reviewers.docx [file pone.0232636.s002.docx]

Re: Response to Reviewers

Manuscript ID: PONE-D-20-01850

Manuscript title: Association Between Statin Use, Atherosclerosis, and Mortality in HIV-infected Adults

April 13, 2020

Dear James M. Wright, Academic Editor, PLOS ONE

On behalf of my co-authors, I thank you for your review of our manuscript and providing us with an opportunity to make revisions. Please find below responses to each of the points raised by the editor and reviewer. Additionally, please find our submitted marked-up copy of our manuscript and a clean unmarked version.

Thank you for providing us with the opportunity to improve our manuscript and make it suitable for publication in PLOS One.

Sincerely,

Binh An P. Phan, MD

Professor of Medicine

University of California, San Francisco

United States

************************************

**Points raised in “Additional Editor Comments”:**

Point #1:

Editor comments:

"In a multivariable Cox survival model with inverse probability treatment weightings, statin users had lower all-cause mortality than non-users but the hazard ratio (HR) was not statistically significant (Table 3)." In the abstract: "All-cause mortality appeared higher in statin users (1.93 per 100 person-years) compared with non-users (1.00 per 100 person-years), but the difference was not significant (adjusted HR=0.74, 95%CI 0.17-3.29, p=0.70)."

Author response:

We agree with this comment noting the conflicting description of overall mortality found in the data table of the results section and what is noted in the abstract. We have corrected the comment in the abstract to be consistent with the data that there was a higher all-cause mortality in the non-statin group that was not significant.

We have corrected the abstract to include the following sentence: “All-cause mortality was higher in the non-statin users compared with statin users, but the difference was not significant (adjusted HR=0.74, 95%CI 0.17-3.29, p=0.07).”

Point #2:

Editor comments:

“Please provide the number of deaths in each group. You report there were 24 overall.”

Author response:

We have included the number of deaths in each group in the results section. There were 3 deaths in the statin users group and 21 deaths in the non-statin users group.

Point #3:

Editor comments:

“In Table 1 the baseline LDL level in the statin group is wrong. You report it as 85. I suspect it must be 105. Please check all the numbers in the manuscript as we may not have identified all of them.”

Author response:

The baseline LDL cholesterol levels were reviewed. The reported median LDL level in Table 1 of 85 mg/dL for the statin group is correct. The average LDL value for this group of statin users was 106 mg/dL. The average and median values are very different for this group as compared to the non-statin group since the number of subjects were small and the LDL levels were skewed due to one subject having very abnormal LDL level. For the non-statin users, the median LDL was 106 mg/dL and average LDL was 112 mg/dL.

Point #4:

Editor comments:

“In the discussion you use the CORONA trial as an example of a trial where statins had no benefit. Please also cite the GISSI-HF trial Lancet 2008, which is a better quality trial showing the same thing.”

Author response:

We agree with this comment and included a sentence noting the similar negative findings from the GISSI-HF and provided a citation.

Point #5:

Editor comments:

“Your findings and other findings in the HIV population suggest to me that a randomized placebo controlled trial is needed in this population.”

Author response:

We thank you for your comment and agree that we need more robust investigation with a randomized controlled trial to help determine the benefits of statin therapy in this increased risk population.

**Journal Requirements:**

When submitting your revision, we need you to address these additional requirements:

1. Please ensure that your manuscript meets PLOS ONE's style requirements, including those for file naming. The PLOS ONE style templates can be found at

http://www.plosone.org/attachments/PLOSOne_formatting_sample_main_body.pdf

http://www.plosone.org/attachments/PLOSOne_formatting_sample_title_authors_affiliations.pdf

Author response:

We have revised our manuscript to meet the PLOS One’s style requirement per the template instructions.

2. Thank you for stating in your Funding Statement:

"This study was funded in part by the National Institute of Allergy and Infectious Diseases (K24AI112393 to PYH). The SCOPE cohort is supported in part also by NIAID (K24AI069994 to SGD), the UCSF/Gladstone Institute of Virology & Immunology (P30AI027763 to SGD), the UCSF Clinical and Translational Research Institute Center (UL1RR024131 to SGD), and the CFAR Network of Integrated Systems (R24AI067039 to SGD).

The funders had no role in study design, data collection and analysis, decision to publish, or preparation of the manuscript."

Please provide an amended statement that declares *all* the funding or sources of support (whether external or internal to your organization) received during this study, as detailed online in our guide for authors at http://journals.plos.org/plosone/s/submit-now. Please also include the statement “There was no additional external funding received for this study.” in your updated Funding Statement.

Please include your amended Funding Statement within your cover letter. We will change the online submission form on your behalf.

Author response:

We have included an amended statement at the end of our cover letter that declares all the funding sources of support received during this study and also have included the updated funding statement that there was no additional external funding received.

3. Please include captions for your Supporting Information files at the end of your manuscript, and update any in-text citations to match accordingly. Please see our Supporting Information guidelines for more information: <http://journals.plos.org/plosone/s/supporting-information>.

Author response:

We have included a Supporting Information and captions at the end of our manuscript and revised our Supporting Information file.

**Review Comments to the Author:**

Reviewer #1: Interesting paper. The paper addressed a relevant question on whether the use of statins has any benefits in decreasing atherosclerosis progression and lowering mortality in patients with HIV infection. The authors did a good job of driving home the fact that some patients may not obtain the expected clinical benefits from statins. Overall, I think the general approach is robust and the findings credible. Appears well carried out. The paper addressed a relevant question but the clinical message is not clear.

To improve the interpretable of the findings, I ask that the authors provide more information on the following points:

Reviewer comment #1:

“The author mentioned that “All-cause mortality appeared higher in statin users (1.93 per 100 person-years) compared with non-users (1.00 per 100 person-years), but the difference was not significant (adjusted HR=0.74, 95%CI 0.17-3.29, p=0.70)” Then, the authors concluded that “Unlike non-HIV infected populations with elevated CVD risk, we found no significant association between statin use and reductions in carotid IMT progression or mortality”

The risks associated with statins use are important to me. Because statins are recommended for general population with high CVD risk, known risks may lead to minimize its use. Death is an outcome that is important to most patients. I wonder if more attention should be paid to screening for the causes of increased mortality among statin users.”

Author response:

We appreciate the comment made by the reviewer and have clarified this discordant statement on mortality as noted above in the editor’s comments. The non-significant increase in mortality was noted in the non-statin group and not the statin group.

Reviewer comment #2:

“In order to clarify the relationship between statin use and all-cause mortality over the 10.9 years of follow up time, it is necessary to carry out a dose-response analysis, which has not been evaluated in this study but which is extremely recommended.”

Author response:

We agree with the reviewer that it would be informative to carry out a dose-response analysis. Unfortunately, due to the small sample size of our study, we did not have power to perform a dose-response analysis and therefore completed our main analysis based upon statin versus non-statin users. We have included the following sentence in the discussion section to highlight this limitation: “A dose-response analysis could not be performed due to the overall small number of statin users.”

Reviewer comment #3:

“What is the median time-to-death between the two groups and was the attributable risk constant over the observation period? It would be helpful to view the cox survival curves (time vs outcome variables).”

Author response:

The number of deaths in each group was small, and therefore we determined that analysis of median time to death was not meaningful. The log-rank test for the Kaplan-Meier survival curves between the statin and non-statin groups was not significant with a p-value of 0.27. We have included a graph of the KM survival curve below for your review. In the results section of the manuscript, we have included the sentence: “The log-rank test for the Kaplan-Meier survival curves between the statin and non-statin groups was not significant (p=0.27).”

Reviewer comment #4:

“The attributable risk difference is small. This means that the finding is sensitive to biases and confounding. While controlling confounding is important to make exposure groups comparable. I have a concern about differential biases. Observational studies cannot account for any unmeasured factor that might influence the outcome. The authors used multiple adjustment strategies. Although the study did not find any benefits from statin use, the study design does not allow to exclude the possibility that the findings may be subject to treatment-selection biases, time related biases and residual confounders by indications that caused unbalance between the two groups. Survivors who were treated with statins could have a worse risk profile for CVD than untreated survivors determined by the pathology rather than by the drugs themselves need to be considered.”

Author response:

We agree with the reviewer that there may be variables that may confound our findings. We used multiple adjustment strategies to minimize bias but understand that other unknown confounders may be present in our observational study. We included this limitation in the discussion of the paragraph.

Reviewer comment #5:

“Please describe the duration of the look-back period for each of the study arms and were there meaningful differences?”

Author response:

The median (IQR) of duration was 3.1 years (1.6, 5.2) for non-statin users and 4.1 years (2.0, 5.0) for statin users. P-value was 0.57, not statistically significant.

Reviewer comment #6:

“The patient involvement in this research was appropriate to its objectives and scope. However, I somehow doubt the conclusions would be broadcast as applying specifically to Caucasian males. Perhaps stressing that even more would help.”

Author response:

We agree with the reviewer. Our study cohort was predominantly male (94%) and Caucasian (61%); which is similar to the demographics of other HIV-infected cohorts in our region. Our findings may not be as applicable to to a female or more diverse HIV-infected population. We have noted that as a limitation of our study and revised it to include the predominant Caucasian representation.
